# The Safety of Radiotherapy in the Treatment of Primary Cutaneous B-Cell Lymphoma: A Multidisciplinary Systematic Review

**DOI:** 10.3389/fonc.2020.01133

**Published:** 2020-07-14

**Authors:** Alessandro Di Stefani, Luca Tagliaferri, Valentina Lancellotta, Bruno Fionda, Barbara Fossati, Mario Balducci, Francesco Federico, Stefan Hohaus, Clara De Simone, Maria Antonietta Gambacorta, Ketty Peris

**Affiliations:** ^1^Institute of Dermatology, Università Cattolica, Rome, Italy; ^2^Fondazione Policlinico Universitario A. Gemelli IRCCS, Rome, Italy; ^3^Dipartimento di Diagnostica per Immagini, Radioterapia Oncologica ed Ematologia, U.O.C. Radioterapia Oncologica, Fondazione Policlinico Universitario A. Gemelli IRCCS, Rome, Italy; ^4^Institute of Pathology, Fondazione Policlinico Universitario A. Gemelli IRCCS, Rome, Italy; ^5^Institute of Pathology, Università Cattolica del Sacro Cuore, Rome, Italy; ^6^Istituto di Ematologia, Università Cattolica del Sacro Cuore, Rome, Italy; ^7^Area di Ematologia, Fondazione Policlinico Universitario A. Gemelli, IRCCS, Rome, Italy; ^8^Istituto di Radiologia, Università Cattolica del Sacro Cuore, Rome, Italy

**Keywords:** primary cutaneous B-cell lymphoma, toxicity, radiotherapy, brachytherapy, multidisciplinary

## Abstract

Primary cutaneous B-cell lymphomas (PCBCL) are rare types of extranodal non-Hodgkin's lymphoma. The choice of treatment usually depends on the variant of PCBCL, number, size, and location of the lesions, involved body surface area as well as patient's age and health condition. The efficacy of radiotherapy (RT) in the treatment of PCBCL has been widely reported conversely, data about the acute and late skin toxicity, patient's treatment satisfaction and quality of life are scarce. A systematic search using PubMed, Scopus, and Cochrane library was performed to identify full original articles analyzing the safety of RT in patients with PCBCL with the primary outcome to assess the acute and late skin toxicity. Secondary outcomes were complete remission, disease free survival, and overall survival. The literature search resulted in 276 articles including eight studies assessing the safety of RT for the treatment of PCBCL. Most patients (median 73%, range 11.9–99.9%) were recorded as having acute skin toxicity of grade 1–2, while acute grade 3–4 toxicity occurred in a median of 8% (range 4–23%) of patients. A median of 20% (range 4–54%) of patients had late skin toxicity of grade 1–2. No late grade 3–4 toxicity was reported. Only one study evaluated patient's satisfaction showing that the 97% of patients were satisfied with radiation therapy. This systematic review confirms the safety of RT in the treatment of PCBCL. Patients with a PCBCL should be managed in highly specialized centers in the context of a multidisciplinary team including dermatologist, hematologist, pathologist, and radiation oncologist.

## Introduction

Primary cutaneous B-cell lymphomas (PCBCL) are rare clinical and histopathological subtypes of extranodal non-Hodgkin's lymphoma (1). The fourth edition of the World Health Organization (WHO) classification system of hematopoietic and lymphoid tumors defined three major PCBCL categories, including: (i) primary cutaneous marginal zone lymphoma (PCMZL), (ii) primary cutaneous follicle center lymphoma (PCFCL), and (iii) primary cutaneous diffuse large B cell lymphoma, leg type (PCDLBCL, LT) (2). The term “PCLBCL, other” or “non otherwise specified” (NOS) encompasses all rare cases of PCBCL not adaptable in the histopathological criteria of the above-mentioned PCLBCL (3). PCBCL have an indolent clinical course in the majority of cases, with 5-year survival rates between 90 and 100% for PCMZL and PCFCL, which are also the most frequent (4–6). Instead, PCDLBCL, LT has a worse prognosis, with 5-year survival rates lower than 50% (4–6). Exclusion of systemic disease is of outermost importance, as PCBCL have a completely different clinical behavior, prognosis, and treatment approach compared with lymphomas characterized by nodal and visceral involvement (4–7). Treatment approaches for PCBCL include surgical excision, radiotherapy (RT), rituximab, and systemic chemotherapy (4–11). As no randomized controlled trials have been performed, treatment recommendations for PCBCL were based on small retrospective studies and single center experiences. To overcome this problem, the European Organization for Research and Treatment of Cancer (EORTC) and International Society for Cutaneous Lymphomas (ISCL), the European Society for Medical Oncology and the International Lymphoma Radiation Oncology Group (ESMO-ILROG) have published consensus treatment recommendations (5, 6, 11). In most cases, optimal patient management requires a multidisciplinary approach, including dermatologists, hematologists, pathologists, and radiation oncologists. RT is considered the most effective treatment for unilesional or localized lesions, with high local control rates and favorable outcome (7–10). Recurrence rates after RT range from 25 to 63% though these data do not differentiate unilesional from multilesional PCBCL (8, 10) and clinical predictive factors for relapse are not yet available.

While the efficacy of RT in the treatment of PCBCL has been widely reported in literature, the acute and late toxicity as well as patient's satisfaction of treatment remained poorly analyzed. Because PCZML and PCFCL are two indolent lymphoproliferative diseases characterized by a good prognosis, it is essential to ensure patient's quality of life and good cosmetic results along with clinical outcome. The present systematic review was performed to primarily assess the safety of RT as treatment of PCBCL.

## Materials and Methods

A systematic search using PubMed, Scopus, and Cochrane library was performed to identify full original articles analyzing the safety of RT in patients with PCBCL. ClinicalTrials.gov was searched for ongoing or recently completed trials, and PROSPERO was searched for ongoing or recently completed systematic reviews. The studies were identified through the following medical subject headings (MeSH) and keywords including “primary cutaneous B cell lymphoma” and “radiotherapy.” The search was restricted to the English language. The Medline search strategy was: “radiotherapy” [Mesh] OR “radiotherapy” [All Fields] AND “primary cutaneous B cell lymphoma” [All Fields]. To avoid missing relevant studies we chose this strategy with high sensitivity but low specificity. We analyzed only clinical full-text studies of PCBCL patients treated with RT. Conference papers, surveys, letters, editorials, book chapters, and reviews were excluded. The time of publication was restricted to the period between 1990 and 2018. Two independent radiation oncologists expert in RT (VL, BRF) screened citations in the titles and abstracts to identify appropriate papers. Eligible citations were retrieved for full-text review. Uncertainties about article inclusion in the review were controlled by an expert multidisciplinary team composed by a radiation oncologist expert in hematological malignancies (MB), a hematologist expert in lymphoproliferative diseases (SH), a pathologist expert in dermatopathology (FF), a dermatologist expert in dermato-surgery (BAF), and a dermatologist expert in skin lymphomas (CDS). Finally, an expert multidisciplinary committee (ADS, LT, MAG, and KP) performed an independent check and the definitive approval of the review.

The primary outcome of our systematic review was the acute and late toxicity. Secondary outcomes included complete remission, disease-free survival, and overall survival. A summary table was created including mono/multicentric studies, sample size, median age, acute and late toxicity, complete remission (CR), disease-free survival (DFS), and overall survival (OS) (Table 1).

**Table 1:** Characteristics of the studies included in the systematic review.

**Reference**	**Period**	**Sample size, *n***	**Median age, years (range)**	**B-cell lymphoma subtype**	**Total dose Gy (range)**	**Median Follow-up months**	**Acute skin toxicity grading**	**Late skin toxicity grading**	**CR**	**DFS at 5 years**	**OS at 5 years**
(12)	2002–2014	39	54 (24–83)	PCMZL 20 pts PCFCL 15 pts PCDLBCL LT 5 pts	30 (4–39.6)	49	G1 33% G2 2.5%		100%		
(13)	1992–2012	42	55 (25–87)	PCFCL 23 pts PCMZL 19 pts	36 (20–46)	113	G1 11.9%		100%	78.8% (95% CI 0.68–0.89)	94.7% (95% CI 0.81–0.99)
(14)	1984–2001	35	61 (27–86)	PCFCL 21 pts PCI 6 pts PCDLBCL LT 3 pts PCBCL NOS 3pts	45 (27–54)	52	G1 85.7% G2 14.2%	G1 8.5%	97%	50% (95% CI 32–68%)	75% (95% CI 55–95%)
(15)	2009–2017	46	51.4 (20–79)	PCFCL 25 pts PCMZL 21 pts	24 (18–30)	43.5	G1 91% G2 9%	G1 21%	96%	51%	100%
(10)	1978–1987	25	50 (23–89)	PCBCL 25 pts	30–40	46.8	G1 92% G2 4% G3 4%	G1 4%	92%	75% (95% CI: 54 ± 97%)	73% (95% CI 51 ± 94%)
(16)	2007–2017	26	55 (21–78)	PCMZL 10 pts PCFCL 16 pts	40 (4–50)	76	G1 61% G2 9%	G1 54%	91%	62%	
(17)	1999–2009	23	53 (26–89)	PCFCL 14 pts PCMZL 7 pts PCDLBCL LT 2 pts	36 (30–44)	40.8	G1 87%	G1 4%	100	71% (95% CI 46–86%)	100%
(18)	1995–2014	86		PCFCL 32 pts PCMZL 20 pts PCBCL NOS 34 pts PCDLBCL, LT	40 (24–46) 40 (4–46) 40 (36–50) 40 (21–46)		G1–G2 69% G1–G2 80% G1–G2 73% —G3–G4 23% G1–G2 67% —G3–G4 8%	G1–G2 28% G1–G2 20% G1–G2 27% G1–G2 8%	63% 65% 73% 67%	61%	92%

*n, number; PCBCL, primary cutaneous B-cell lymphomas; PCMZL, primary cutaneous marginal zone lymphoma; PCFCL, primary cutaneous follicle center lymphoma; PCBCL NOS, PCBCL non otherwise specified; PCI, primary cutaneous immunocytoma; PCDLBCL, primary cutaneous diffuse large B-cell lymphoma of the leg; Gy, gray; CR. Complete remission; DFS. Disease free survival; OS, overall survival*.

## Results

**Figure 1:**
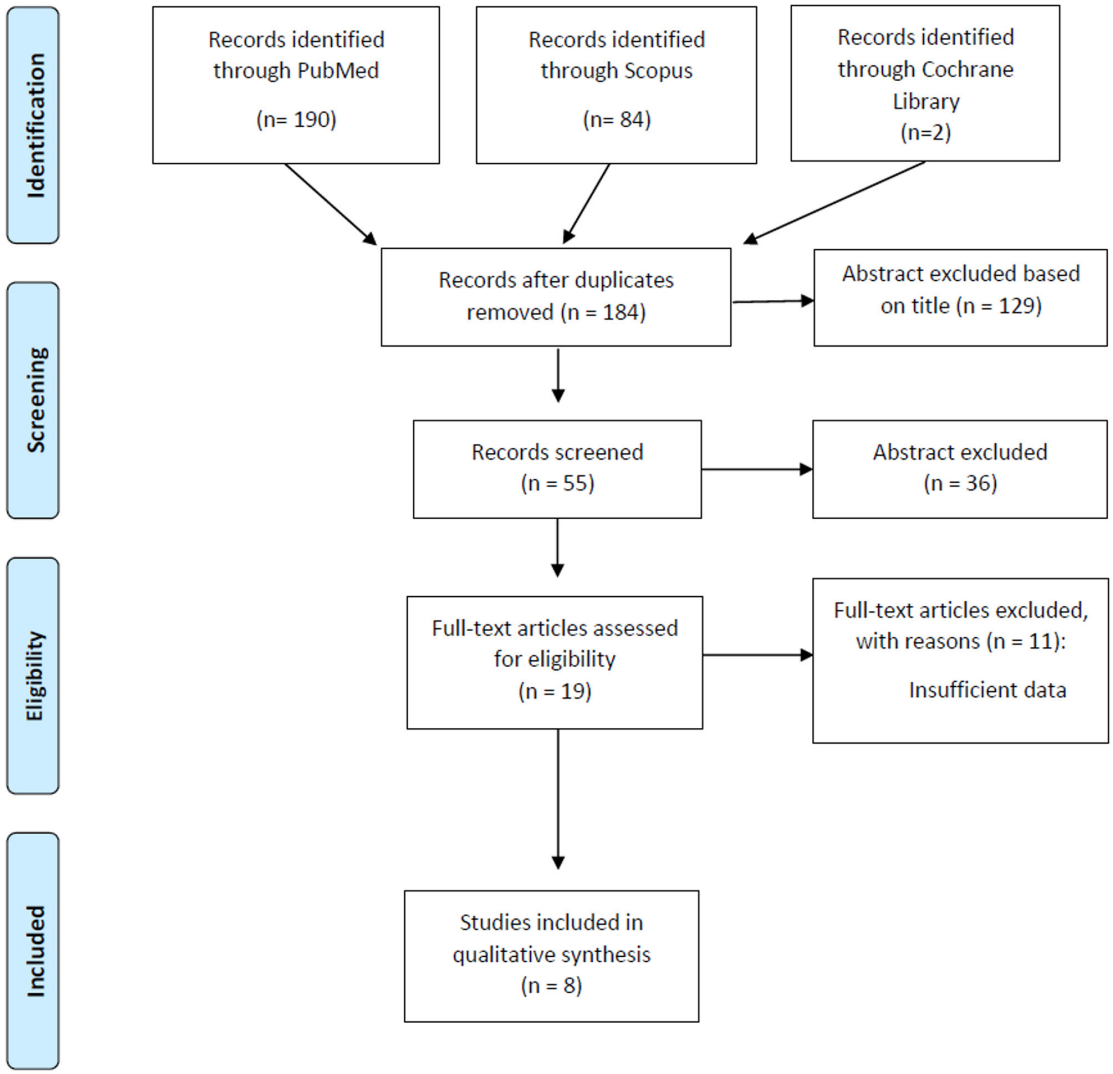
PRISMA flow-chart for outcome and late adverse effects.

The literature search resulted in 276 articles. After exclusion on the basis of the title and abstract, and exclusion of conference papers, surveys, letters, editorials, book chapters, reviews, and language other than English, 19 papers were assessed via full text for eligibility. Of these, 11 articles were excluded because of insufficient data, while eight studies were considered adequate to evaluate the safety of RT for the treatment of PCBCL (Figure 1). All studies were retrospective (10, 12–18). In accordance with the selection criteria, only data from the RT toxicity arms were extracted and considered for the analysis. Table 1 lists the characteristics of the studies included in our review. We identified 322 patients (median age: 54 years), 97 of whom were affected by PCZML, 146 by PCFCL, 44 by PCDLBCL, LT, and 35 were PCBCL non otherwise specified (NOS). All patients underwent external beam RT. Multiagent systemic treatment was reported in five of the eight studies. The median proportion of patients treated with chemotherapy before RT was 21% (range 12–52%). Surgery was performed in 15 patients (4.6%). External beam RT was delivered with a median total dose of 36 Gy (range 4–54 Gy). The median follow-up was 49 months (range 40.8–113 months).

Most patients (median 73%, range 11.9–99.9%) were recorded as having acute skin toxicity of grade 1–2 (consisting in erythema, xerosis, and itching), while acute skin toxicity of grade 3–4 occurred in a median of 8% (range 4–23%) of patients (consisting in exudative dermatitis, erosions, and skin necrosis). A median of 20% (range 4–54%) of patients experienced late grade 1–2 skin toxicity (consisting in mild pigmentation changes, atrophy, telangiectasis, and hair loss in the irradiation field). No late grade 3–4 toxicity was observed. A single study compared conventional-dose RT to low-dose RT in terms of acute toxicities showing a lower incidence of grade 1 and grade 2 in de-escalated RT approach (70 vs. 14% grade 1; 8 vs. 0% grade 2; *p* = 0.004) (16).

Just one study reported patient's satisfaction of cosmetic results by an oral standardized questionnaire showing that 35/36 patients answered to be satisfied by radiation therapy; the only patient resulted unsatisfied due to acute toxicity and late sequelae (15).

The median CR rate following RT was 92% (range 63–100%). Relapse occurred in 34.7% of patients (29.5% outside the radiation field and 5.2% in field). DFS at 5 years was 62% (range 50–78.8%) and the OS at 5 years was 93.5% (range 73–100%).

## Discussion

The efficacy of RT in the treatment of PCBCL has been widely reported in literature, however, the safety as well as patient's satisfaction remained poorly analyzed. The main aim of our systematic review was to evaluate the acute and late toxicity of RT in the treatment of PCBCL. The present systematic review shows the safety of RT with the most common toxicity being grade 1–2 acute skin toxicity.

Several therapeutic modalities are available for PCBCL and consensus treatment recommendations usually depends basically on the type of PCBCL, the size, and location of the lesions In patients with solitary or localized lesions of PCDLBCL, LT, or PCLBCL NOS, the first line treatment is the R-CHOP (rituximab, cyclophosphamide, adriamycin, vincristine, and prednisone) regimen, followed by local RT. If the patient does not tolerate chemotherapy, RT alone or RT combined with rituximab may be used (11, 15–18). In PCMCL and PCFCL patients with solitary skin lesions, low-dose radiation therapy is safe and effective, with a complete remission rate approaching 100% (19–22). Radiation therapy does not appear to be inferior to multiagent chemotherapy also in patients with multiple lesions and no differences in relapse rate and long-term DFS were reported between patients treated with surgery or RT (21–23).

The indolent clinical behavior of PCMZL and PCFCL along with the rarity of extracutaneous relapses make local radiation a valid treatment option, nevertheless in clinical practice the management continues to include local surgical resection or systemic chemotherapy (4). Many studies showed that cancer specific survival for PCMZL and PCFCL is close to 100%, and DFS for solitary lesions is 77% indicating that too aggressive treatments are not necessary (24). Sometime, treatment choice is based not only on patient- and disease-related factors but is also influenced in the single center by issues related to equipment availability and institutional-based experiences (25). RT of PCBCL is a highly individualized treatment and has been demonstrated to be extremely effective in all subtypes. Since the decision of the dose and field size is highly dependent on the histologic subtype and extent of the skin involvement as well as on previous treatments, the type of RT technique plays a crucial role in achieving optimal outcome. Most frequently applied local RT method is the use of multiple energies of electron beams (26). However, there are also technical issues influencing the choice of the preferred RT technique. External beam radiation represents the most common application but in selected cases interventional RT, also called brachytherapy, in form of an individual surface mold, could be a better solution. The major differences between these two RT technologies are based on technical and physical details of the optimal application of the right dose to the right target. From a physical point of view, the homogenous irradiation of highly irregular clinical targets on the surface of the body, like hand, fingers, foot, or full face is not always possible with electron/photons beams, but well applicable with individual surface molds. From a technical point of view, optimal target coverage and highly conformal dose distribution are applicable without numerous field matchings or complex bolus applications. The use of the potential of individual dose intensity modulation by the stepping source technology combined with individual CT-based dose-volume optimization results in excellent dosimetric coverage of complex superficial targets, also in large and individually shaped volumes (27, 28).

Surface mold brachytherapy is rarely reported in the treatment of cutaneous lymphomas, however, the successful use of this technology is common and frequently reported in the treatment of other cutaneous tumors such as non-melanoma skin cancers (29–41). Interventional RT may be proposed as the treatment of choice for elderly patients with poor performance status and/or severe co-morbidities, due to its relatively short total treatment time. Interventional RT avoids the difficulties associated with age-related loss of mobility, patient positioning, and set-up procedures (42, 43). Although most of the recently published review papers advise to apply local RT for PCBCL, none of them discusses the application of surface molds (5, 6, 11).

Patients with PCBCL have an excellent OS, therefore the preservation of quality of life and the reduction of toxicity should be the main treatment goals, and should be also considered as outcome measures in clinical trials comparing treatment modalities. Few studies analyzed the acute and late skin toxicity after RT. Our systematic review showed that RT is safe in the treatment of PCBCL: although acute skin toxicity occurred in the great majority of the patients, it was mild, rarely exceeding grade 1–2, and consisted mostly in erythema, dry skin, and pruritus. As well, grade 1–2 late skin toxicity was observed in about 20% of patients essentially in the form of dyschromia, more rarely atrophy, telangiectasis, and alopecia in the irradiation field. The type of skin toxicities resulted consistent in all included studies. Moreover, no cases of secondary skin cancers in the radiation field were reported. Literature data reported a wide variation in toxicity rates and a correct interpretation is further complicated by the various clinical scoring criteria used in different studies like radiation therapy oncology group (RTOG) or common terminology criteria for adverse events (CTCAE). Moreover, no studies explored the correlation between RT toxicity rate and previous treatments, probably due to the low proportion of patients treated by chemotherapy and/or surgery before RT. Finally, only one study out of eight analyzed the association of RT total dose with the incidence and severity of skin toxicity highlighting a reduced rate in the low-dose RT (16). Remarkably, effective local control has been achieved even with doses as low as 4 Gy, especially in indolent PCBCL (12).

Patient's satisfaction with treatment results is still a rarely analyzed endpoint in PCBCL. A single study evaluated patient's opinion about cosmetic result of RT showing that in almost the totality of patients skin tolerance of RT was considered satisfactory for the excellent long-term cosmetic outcome. The only patient who was unsatisfied, indeed complained of acute toxicity and late sequelae (15).

The paucity of studies on this topic highlights the importance of a multidisciplinary team including dermatologist, hematologist, pathologist, surgeon as well as radiation oncologist, and interventional radiotherapist for a more individualized management of PCBCL patients (44). The pivotal role of a multidisciplinary setting was recently evidenced in a study on patients with esophageal cancer demonstrating the independent influence of the hospital of diagnosis on the probability to receive curative treatment (45). It is important to increase awareness regarding PCBCL treatment, particularly emphasizing the favorable clinical course and excellent cosmetic outcomes after interventional RT, in order to reduce the risk of overtreatment with high doses of RT or systemic chemotherapy. The possibility to identify one subgroup of PCBCL (i.e., PCMZL and PCFCL) with indolent behavior should be used for the selection of patients eligible to interventional RT ensuring safe treatment and better quality of life. A combined analysis of treatment results from different centers is highly needed in order to create predictive models (43, 46–56).

In conclusion, this systematic review confirms the safety of RT in the treatment of PCBCL with an excellent long-term cosmetic result for almost all patients associated with high patient's satisfaction and good quality of life. Patients with a PCBCL should be managed in centers where a close collaboration between dermatologists, hematologists, pathologists, and radiation oncologists exists.

## Data Availability Statement

All datasets analyzed for this study are included in the article/supplementary material.

## Author Contributions

Conception and design: AD, LT, MG, and KP. Data collection: VL, BFo, CD, BFi, FF, SH, and MB. Manuscript writing: AS, VL, and LT. Analysis and interpretation of data: AS, LT, MG, and KP. All authors contributed to the article and approved the submitted version.

## Conflict of Interest

The authors declare that the research was conducted in the absence of any commercial or financial relationships that could be construed as a potential conflict of interest.
